# Third Congress for the European Society for Emerging Infections

**DOI:** 10.3201/eid1104.041297

**Published:** 2005-04

**Authors:** Maria Mavris, Lénaïg Halos

**Affiliations:** * Ecole Nationale Veterinair d'Alfort, Maisons-Alfort, France

**Keywords:** Infectious diseases, zoonoses, emerging

**Third Congress for the European Society for Emerging Infections**

Ecole Nationale Vétérinaire d'Alfort in Maisons-Alfort, France

October 2004

The Third Congress for the European Society for Emerging Infections brought together an impressive list of scientists, clinicians, and veterinarians from different fields, including Nobel Prize winner Professor Carlton Gadjusek, and encouraged a dialogue related to emerging infections. The theme of this meeting was that, because many infectious diseases have a zoonotic origin, disease processes in animals should be examined, not just considered as models for humans.

Various strategies used by viruses and bacteria to infect their hosts and subsequently persist were discussed. Deaths attributable to infectious diseases are ≈25% of the total number of human deaths worldwide annually. Changes in demographics and behavior are the main influences on the emergence of new diseases. Many existing and emerging diseases arose from animals: 75% of new human diseases are zoonotic in origin. Changes that affect the pathogen spectrum include climatic factors, such as global warming, increased volume and speed of international travel, increased trade, deforestation, urbanization, and adaptation of microbes to new environments.

An example of microbe environmental adaptation is the discovery of an African mosquito-borne flavivirus in central Europe. Large numbers of blackbirds and owls in the zoological gardens and around Vienna, Austria, had been found dead in the summer of 2001. Upon further investigation, they were found to have swollen livers and spleens as well as other characteristics of West Nile virus. Reverse transcription–polymerase chain reaction (RT-PCR) determined that the flavivirus was Usutu virus from South Africa, which had been carried to Europe by migratory swallows. Some spread has occurred to the 4 countries bordering Austria; however, no human clinical cases have occurred to date.

On a global scale, the most important vector-borne infections are mosquito-transmitted, whereas in Europe tick-transmitted infections are a major concern. An example of a tickborne infection is ehrlichiosis. This disease was first described in the United States in 1994 but was described in animals in Europe in the 1960s. At least 20 cases in humans have now been reported in Europe. *Anaplasma phagocytophilum*, the causative agent, can be detected by PCR in 2% to 45% of *Ixodes ricinus* ticks. Animals can act as good sentinels for human infection. Erhlichiosis due to *A. phagocytophilum* may also be associated with a high risk of co-infection by *Coxiella*, *Babesia*, and *Theileria*. Therefore, information and education for both veterinarians and general practitioners are important for managing and identifying this widespread zoonosis. The combined broad-range PCR-TTGE (temporal temperature gradient gel electrophoresis) technique can detect several bacterial pathogens in ticks in a single step.

Transmissible spongiform encephalopathies, in particular variant Creutzfeldt-Jakob disease (vCJD), are caused by prions, which are able to cross species barriers. Relatively little is known about their routes of transmission. Recent experiments for vCJD have shown that the intravenous route for infecting Macaque monkeys is as efficient as the previously used intracerebral route, which implies a risk for transmission through blood transfusion. Indeed, 2 cases due to blood transfusions have been identified in humans. Risk factors for vCJD include dietary exposure to the infectious agent of bovine spongiform encephalopathy and may be related to the low mean age (29 years) for death due to this disease. In addition, many patients with this disease live relatively close to each other; however, the implications of this finding are unclear.

The meeting concluded with the topical subject of emerging respiratory diseases, in particular those caused by coronaviruses and influenza viruses. The host range of coronaviruses is greater than initially thought. Feline and canine coronaviruses are closely related to porcine gastroenteritis virus and are also able to replicate and cause enteric disease in swine. Coronavirus relatedness is well demonstrated by the coronavirus associated with severe acute respiratory syndrome (SARS). Like many of the new emergent diseases, SARS has a zoonotic origin that is as yet unknown. A total of 8,098 cases of SARS and 774 deaths (case-fatality rate of 9.6%) have occurred worldwide; the incidence is higher among healthcare workers (case-fatality rate 17%). The spread of SARS was due to global travel and was also attributed to illegal wildlife trade. Delays in identifying symptoms or clusters of case-patients increased the risk of contracting SARS, especially in healthcare workers, and inadequate personal protective equipment played a large role in its spread.

The need for global medical and veterinary information systems was emphasized. Strict adherence to protective measures for avoiding bird-to-human transmission, in the context of avian influenza viruses, was also underscored, particularly since the Asian outbreak, which began in December 2003, is still ongoing.

In general, the conference encouraged participants to exchange ideas and to consider future collaborations. One suggestion for coordinating such efforts could be a European version of the Centers for Disease Control and Prevention.

In 1969, then-Surgeon General William H. Stewart stated that "the time has come to close the book on infectious diseases," demonstrating a misinformed and rather optimistic opinion. By contrast, the third congress ended with a warning "to expect the unexpected."

